# Features of *GBA*-associated Parkinson’s disease at presentation in the UK *Tracking Parkinson’s* study

**DOI:** 10.1136/jnnp-2017-317348

**Published:** 2018-01-29

**Authors:** Naveed Malek, Rimona S Weil, Catherine Bresner, Michael A Lawton, Katherine A Grosset, Manuela Tan, Nin Bajaj, Roger A Barker, David J Burn, Thomas Foltynie, John Hardy, Nicholas W Wood, Yoav Ben-Shlomo, Nigel W Williams, Donald G Grosset, Huw R Morris

**Affiliations:** 1 Department of Neurology, Ipswich Hospital NHS Trust, Ipswich, UK; 2 Department of Molecular Neuroscience, UCL Institute of Neurology, London, UK; 3 Institute of Psychological Medicine and Clinical Neurosciences, MRC Centre for Neuropsychiatric Genetics and Genomics, Cardiff University, Cardiff, UK; 4 School of Social and Community Medicine, University of Bristol, Bristol, UK; 5 Department of Neurology, Institute of Neurological Sciences, Queen Elizabeth University Hospital, Glasgow, Scotland; 6 Department of Clinical Neuroscience, UCL Institute of Neurology, London, UK; 7 Department of Neurology, Queen’s Medical Centre, Nottingham, UK; 8 Department of Clinical Neurosciences, John van Geest Centre for Brain Repair, Cambridge, UK; 9 Faculty of Medical Sciences, University of Newcastle, Newcastle upon Tyne, UK; 10 Sobell Department of Motor Neuroscience, UCL Institute of Neurology, London, UK; 11 Department of Molecular Neuroscience, Reta Lila Weston Laboratories, UCL Institute of Neurology, London, UK

## Abstract

**Objectives:**

To examine the influence of the glucocerebrosidase (*GBA*) mutation carrier state on age at onset of Parkinson’s disease (PD), the motor phenotype and cognitive function at baseline assessment in a large cohort of UK patients. We also analysed the prevalence of mood and behavioural problems that may confound the assessment of cognitive function.

**Methods:**

We prospectively recruited patients with PD in the *Tracking Parkinson’s* study. We fully sequenced the *GBA* gene in all recently diagnosed patients (≤3.5 years). We examined cognitive (Montreal Cognitive Assessment) and motor (Movement Disorder Society Unified Parkinson’s Disease Rating Scale part 3) function at a baseline assessment, at an average of 1.3 years after diagnosis. We used logistic regression to determine predictors of PD with mild cognitive impairment and PD with dementia.

**Results:**

We studied 1893 patients with PD: 48 (2.5%) were heterozygous carriers for known Gaucher’s disease (GD) causing pathogenic mutations; 117 (6.2%) had non-synonymous variants, previously associated with PD, and 28 (1.5%) patients carried variants of unknown significance in the *GBA* gene. L444P was the most common pathogenic *GBA* mutation. Patients with pathogenic *GBA* mutations were on average 5 years younger at disease onset compared with non-carriers (P=0.02). PD patients with GD-causing mutations did not have an increased family risk of PD. Patients with *GBA* mutations were more likely to present with the postural instability gait difficulty phenotype compared with non-carriers (P=0.02). Patients carrying pathogenic mutations in *GBA* had more advanced Hoehn and Yahr stage after adjustment for age and disease duration compared with non-carriers (P=0.005). There were no differences in cognitive function between *GBA* mutation carriers and non-carriers at this early disease stage.

**Conclusions:**

Our study confirms the influence of *GBA* mutations on the age of onset, disease severity and motor phenotype in patients with PD. Cognition did not differ between *GBA* mutation carriers and non-carriers at baseline, implying that cognitive impairment/dementia, reported in other studies at a later disease stage, is not present in recently diagnosed cases. This offers an important window of opportunity for potential disease-modifying therapy that may protect against the development of dementia in *GBA*-PD.

**Clinical trial registration:**

NCT02881099; Results.

## Introduction

Initial reports of an association between some types of Gaucher’s disease (GD) and parkinsonism led to the investigation of the link between heterozygous mutations in the gene coding for the enzyme glucocerebrosidase (*GBA*) and Parkinson’s disease (PD).^1 2^ *GBA* mutations are common in PD and are the most important risk factor yet discovered for PD.^3^ A multicentre study of *GBA* mutation carriers, spanning three continents, established that the OR for any *GBA* mutation in patients with PD versus controls without PD was about 5.4.^4^ Variants in *GBA* can influence the phenotype of PD.^5^ Mutations that are not commonly recognised as causing GD have been found in patients diagnosed with PD^6^ and in particular the *GBA* E326K variant may predispose to PD.^7^ As compared with patients who do not carry a *GBA* mutation, those with *GBA* mutations have been reported to present earlier, are more likely to have affected relatives and are more likely to have atypical clinical manifestations.^4^ Furthermore, mild and severe heterozygous *GBA* mutations can differentially affect the risk and the age at onset of PD.^8^ *GBA* mutation carriers with PD are on a trajectory to cognitive decline^9^ despite clinicopathological studies showing no statistically significant difference in Braak stages between *GBA* mutation carriers and sporadic PD controls.^10^

Motor progression rates differ in *GBA* mutation carriers with PD. The HR for progression to Hoehn and Yahr stage 3 is significantly greater in *GBA* mutation carriers with PD compared with non-carriers.^11^ There is also evidence to suggest that carriers of polymorphisms in *GBA* which are not generally considered to increase PD risk are at significantly increased risk of progression to Hoehn and Yahr stage 3.^11^ More recent data from a prospective longitudinal study suggest that *GBA*-associated patients with PD not only have more rapid progression of disease but also have reduced survival.^12^

Cognitive impairment is a common problem in the later stages of PD,^13^ but even in early-stage disease there are mild deficits that can be identified on formal cognitive testing.^14^ Cognitive impairment in PD exists on a spectrum from mild cognitive impairment in PD (PD-MCI) to dementia (PD-D). Further, some types of PD-MCI may be a harbinger of dementia which occurs in up to 80% of patients over 15–20 years.^13^ and recent studies have identified several genetic factors that influence this including *ApoE, MAPT* and *SNCA*.^14 15^ *GBA* mutation status is reported as an independent risk factor for cognitive impairment in patients with PD.^16 17^ Further, *GBA* variants are reported to associate with a distinct pattern of cognitive deficits in PD characterised by greater impairment in working memory, executive function and visuospatial abilities.^18^ The individual risk of dementia in PD patients with *GBA* mutations is increased sixfold in carriers compared with non-carriers.^17^ The HRs for progression both to dementia and Hoehn and Yahr stage 3 are significantly greater in *GBA* mutation carriers compared with non-carriers, and in fact mutations in *GBA* have also been found in cases with dementia with Lewy bodies (DLB), thus pointing towards a link between *GBA*, parkinsonism and dementia.^19^

This collection of evidence from smaller studies linking *GBA* mutation status to several clinical phenotypic characteristics including disease progression and survival in PD has implications for genetic counselling, clinical follow-up and stratification in future clinical trials.

We assessed the influence of *GBA* mutations on the age of onset of PD, early motor phenotype, motor staging and cognitive function in a very large cohort of patients (n>2000, figure 1).

**Figure 1:**
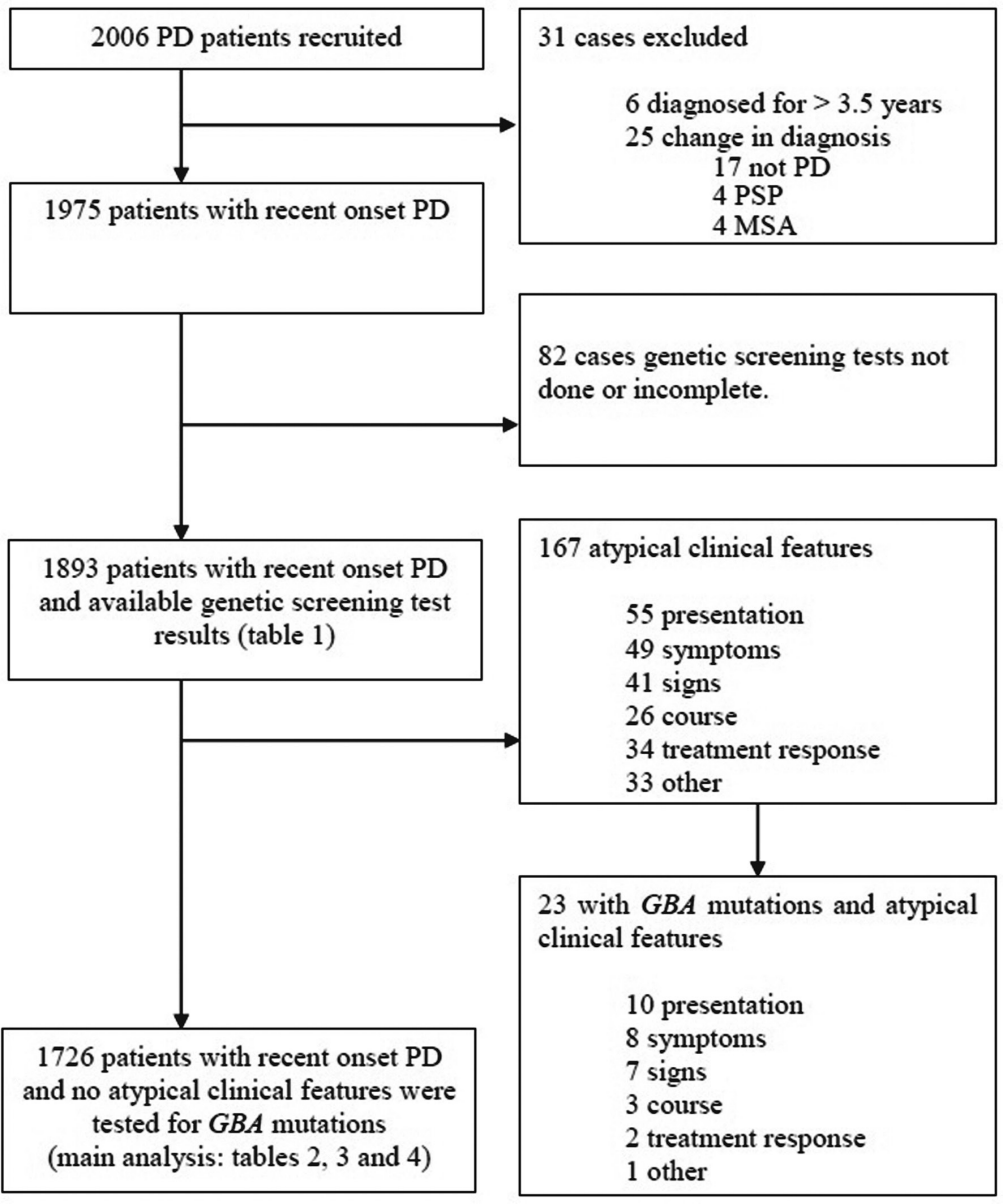
Patient recruitment and assessment in this study. The main analysis of cognitive function using the Montreal Cognitive Assessment was performed in cases without features that might be atypical for Parkinson’s disease (PD). GBA, glucocerebrosidase PSA, Progressive supranuclear palsy, MSA, multiple system atrophy.

## Methods

*Tracking Parkinson’s* is a large prospective, observational, multicentre project in the UK. The study set-up and design have been previously reported.^20^ Patients were recruited with a clinical diagnosis of PD, fulfilling Queen Square Brain Bank criteria^21^ and supported by structural and/or functional neuroimaging. Both drug-naive and treated patients aged 18–90 years were eligible. Recent onset cases were diagnosed with PD in the preceding 3.5 years, and recruitment was completed between February 2012 and May 2014. Patients with a clinical diagnosis of dementia at their first assessment were excluded. Enrolled patients whose diagnosis was later changed, on clinical or imaging grounds, were excluded from analysis. Additionally, patients with atypical features, which might indicate an alternative diagnosis, were excluded from the data analysis (figure 1).

Motor subtypes were determined from the Movement Disorder Society Unified Parkinson’s Disease Rating Scale part 3 (MDS-UPDRS 3) scores using a predetermined formula.^22^ The Montreal Cognitive Assessment (MoCA) test scores were adjusted for years of education and predetermined diagnostic cut-offs were used, to categorise cases into normal (>23), MCI 22–23 and dementia (<22), matching previous similar studies.^20 23^ Depression and anxiety were identified from scores >6 in the Leeds Hospital Anxiety and Depression Scale.^24^ Rapid eye movement (REM) sleep behaviour disorder (RBD) was defined as a score >4 on the RBD screening questionnaire.^20^ Olfaction testing was performed using either the 40-item University of Pennsylvania Smell Identification Test or Sniffin’ Sticks 16-item version, and hyposmia was defined as previously reported.^25^

### Molecular genetic analysis of GBA mutations

The coding exons of *GBA* were screened using a nested PCR protocol followed by DNA sequencing described by Neumann *et al*.^10^ In accordance with the established convention for *GBA* alleles, all genetic variants were named according to the processed protein excluding the 39 residue signal peptide. Genetic variants were classified according to the criteria suggested by Winder-Rhodes *et al* 11; *GBA* mutations that had been previously identified as being pathogenic in GD and associated with PD in the heterozygous state (group 1: e.g. L444P and G370S), non-synonymous genetic variants that have been linked to GD when occurring with other *GBA* mutations and have been associated with PD (group 2: eg, E326K and T369M) and genetic variants of unknown significance (group 3). Cases were then classified into one of these three groups depending on their *GBA* mutation status. Individuals with no sequence variants were classed as ‘non-carriers’ and combined with individuals from group 3. Further, analyses were performed comparing groups 1 and 2 with non-carriers of *GBA* mutations (group 3). Subgroup analyses were subsequently performed to compare cognition in p.L444P carriers to non-carriers and p.L444P mutation carriers to p.N370S carriers. Additional analyses were also performed to compare cognition in those with atypical features who carried *GBA* mutations and non-carriers.

### Statistical analyses

Demographic and phenotypic characteristics were analysed using multivariable regression. Analyses of continuous variables (where UPDRS 3 and Parkinson’s disease questionnaire 8 items (PDQ8) scores were assumed continuous) were carried out using linear regression and binary variables using logistic regression. For MoCA scores, standard linear regression was used; for individual MoCA domains, ordered logistic regression was used. Where a category had small numbers (<3% of total), it was merged with the category above to ensure stability. Comparisons of Hoehn and Yahr stages were also carried out with ordered logistic regression. Comparisons of motor subtypes were performed using multinomial logistic regression with tremor dominant group as the comparator.

All P values were two-tailed; P values were calculated after adjustment for potential confounders where the confounders are listed in each table. The linearity of continuous confounders (such as age and disease duration) was tested using fractional polynomials in univariate models and then transformed if they showed evidence of non-linearity. We tested the proportional odds assumption in all ordered logistic regression models (except supplementary subgroup analyses with smaller numbers). All of these passed except within three models, and relaxing the model to a non-proportional odds model made no qualitative difference to the results.

Finally, we performed additional imputed analyses due to some missing data in the years of education (n=107), in the full MoCA test questionnaire (n=140) and within individual MoCA domains (varying between n=22–67 missing data elements). This involved using the chained equation approach to multiple imputation, creating 10 imputed datasets, then deriving estimates and P values with Rubin’s rules.

Statistical analyses were performed using STATA V. 13 (StataCorp).

## Results

A total of 5 different pathogenic mutations, 28 variants of unknown significance (including two exon deletions) and 2 PD-associated non-GD variants (E326K, T369M) were found among 1893 patients with PD. Of these 1893 cases, 48 (2.5%) carried one or more GD pathogenic mutations; all of these cases were simple heterozygotes. In total, 117 carried PD-associated variants: 86 (4.5%) carried E326K mutation(s) (one case was homozygous for this mutation; the rest were heterozygous), 35 (1.8%) carried the T369M mutation (table 1) and 1728 were non-carriers of *GBA* mutations. Four carried more than one PD-associated variant. There were 167 cases with atypical features: 144 (86.2%) were non-carriers but 23 (13.8%) tested positive for *GBA* mutations. Of these 23 cases, 4 (2.4%) showed GD pathogenic variants while 19 (11.4%) had PD-associated non-GD variants (E326K or T369M). Detailed comparisons of phenotypic characteristics (tables 2–4) were performed on those without atypical clinical features that could raise suspicion of an alternative diagnosis (n=1726) (figure 1).

**Table 1:** *GBA* variants found in the 1893 recently diagnosed patients from the *Tracking Parkinson’s* study

Cases, n (%)	Recognised GD pathogenic mutations	PD-associated non-GD variants	Rare variants of unknown significance
30 (1.6%)	p.L444P		
11 (0.6%)	p.N370S		
5 (0.3%)	p.R463C		
2 (0.1%)	p.G202R		
2 (0.1%)	p.R359S		
86 (4.5%)		p.E326K	
35 (1.8%)		p.T369M	
26* (1.4%)			p.D409H, p.F213I, p.G189V, p.G377S, p.K157Q, p.L383Xfs, p.L66P, p.M123T, p.N382Xfs, p.R163s, p.R257Q, p.S173s, p.E481Xfs, p.G10S, p.G325W, p.R170H, p.T323I, p.L175I, p.L324V, p.P55S, p.R262H, p.R329H p.R395C, p.T267I, p.L268L, Asp315His, Exon 3 hemizygous deletion
7† (0.4%)			p.A456P, p.V460V
6‡(0.3%)			p.D140H, p.I308T, Ex4 hemizygous deletion

*Each of the 27 variants was found in single cases from our cohort, although one individual had two of these variants (total n=26).

†Each variant was found in six cases from our cohort, although some individuals had more than one of these variants (total n=7).

‡Each variant was found in two cases from our cohort (total n=6).

GD, Gaucher’s disease; PD, Parkinson’s disease.

**Table 2:** Demographic and clinical features of the Parkinson’s disease (PD) cohort classified by *GBA* mutation carrier status

Variable	GD-causing variants (group 1) n=44	E326K and T369M carriers (group 2) n=98	Non-carriers (group 3) n=1584	P value* Group 1 vs 3	P value* Group 2 vs 3	P value* Groups 1 and 2 vs 3
Age (years)	62.9 (12.3)	66.8 (8.7)	67.6 (9.2)	<0.001†	0.50†	0.018†
Age at diagnosis (years)	61.4 (12.3)	65.6 (8.7)	66.2 (9.2)	<0.001†	0.50†	0.018†
Duration from diagnosis (years)	1.5 (1.1)	1.2 (0.9)	1.3 (0.9)	0.15‡	0.075‡	0.49‡
Gender, male (%)	28 (63.6%)	65 (66.3%)	1036 (65.4%)	0.93§	0.83§	0.90§
HY stage						
0–1.5 (%)	17 (38.6%)	48 (49.0%)	774 (49.3%)	0.015	0.81	0.14
2 or 2.5 (%)	21 (47.7%)	47 (48.0%)	704 (44.9%)			
3+ (%)	6 (13.6%)	3 (3.1%)	91 (5.8%)			
UPDRS 3	24.2 (13.0)	21.8 (11.5)	22.6 (12.2)	0.26¶	0.72¶	0.76¶
LEDD (mg/day)	370 (219)	303 (183)	292 (206)	0.028	0.17	0.020
Education≤12 years (%)	14 (34.1%)	29 (31.2%)	483 (32.5%)	0.47	0.91	0.78
Motor subtype						
TD	12 (30.0%)	38 (41.3%)	688 (47.7%)			
PIGD	20 (50.0%)	41 (44.6%)	566 (39.3%)	0.061	0.14	0.027
Indeterminate	8 (20.0%)	13 (14.1%)	187 (13.0%)	0.057	0.44	0.10
Anxiety (%)	14 (33.3%)	24 (25.3%)	355 (23.5%)	0.35	0.76	0.44
Depression (%)	10 (24.4%)	27 (28.1%)	338 (22.3%)	0.98	0.20	0.28
QOL score	6.2 (4.9)	6.0 (5.0)	5.7 (4.7)	0.91	0.42	0.46
Family history of PD						
First degree (%)	5 (11.4%)	8 (8.2%)	194 (12.3%)	0.82	0.20	0.24
Second degree (%)	6 (13.6%)	4 (4.1%)	155 (9.9%)	0.55	0.063	0.23
Any (%)	9 (20.5%)	12 (12.2%)	319 (20.3%)	0.87	0.048	0.087
Ethnicity						
White	44 (100%)	98 (100%)	1531 (98.0%)	NA	NA	NA
Asian or Asian British	–	–	15 (1.0%)	–	–	–
Black or black British	–	–	12 (0.8%)	–	–	–
Mixed	–	–	3 (0.2%)	–	–	–
Others	–	–	2 (0.1%)	–	–	–
RBD symptoms (%)	17 (43.6%)	46 (49.5%)	638 (43.1%)	0.92	0.20	0.31
Olfactory loss	33 (86.8%)	69 (80.2%)	968 (71.0%)	0.047	0.062	0.009

Data are shown as mean and SD unless expressed otherwise.

*Adjusted for age, gender and disease duration (except where otherwise noted).

†Adjusted for gender and disease duration.

‡Adjusted for gender and age.

§Adjsuted for age and disease duration.

¶Adjusted for age, gender, disease duration and LEDD.

GD, Gaucher’s disease; HY, Hoehn and Yahr stage; LEDD, levodopa-equivalent daily dose; NA, not available; PIGD, postural instability gait difficulty; QOL, quality of life based on PDQ8 scale; RBD, rapid eye movement sleep behaviour disorder; TD, tremor dominant; UPDRS 3, Movement Disorder Society Unified Parkinson’s Disease Rating Scale part 3.

**Table 3:** Cognitive impairment in the *GBA*-associated Parkinson’s disease cohort classified by subdomain based on Montreal Cognitive Assessment (MoCA)

Variable (range of possible scores)	GD-causing variants (group 1) n=44	E326K and T369M carriers (group 2) n=98	Non-carriers (group 3) n=1584	OR* (95% CI) Group 1 vs 3	P value* Group 1 vs 3	OR* (95% CI) Group 2 vs 3	P value* Group 2 vs 3	OR* (95% CI) Groups 1 and 2 vs 3	P value* Groups 1 and 2 vs 3
Visuospatial (0–5)	4.2 (1.1)	4.2 (1.1)	4.3 (1.0)	1.27 (0.68 to 2.40)	0.45	1.09 (0.71 to 1.66)	0.70	1.14 (0.80 to 1.63)	0.48
Attention (0–6)	5.3 (0.9)	5.2 (1.0)	5.3 (1.0)	1.21 (0.66 to 2.22)	0.55	1.28 (0.86 to 1.90)	0.23	1.25 (0.89 to 1.76)	0.19
Language (0–3)	2.6 (0.5)	2.3 (0.7)	2.4 (0.8)	0.80 (0.42 to 1.51)	0.49	1.40 (0.93 to 2.12)	0.11	1.19 (0.84 to 1.68)	0.34
Naming (0–3)	2.8 (0.4)	2.9 (0.3)	2.9 (0.3)	3.18 (1.32 to 7.67)	0.010	1.01 (0.45 to 2.25)	0.99	1.53 (0.84 to 2.78)	0.17
Recall (0–5)	2.8 (1.7)	2.7 (1.6)	2.6 (1.6)	1.09 (0.60 to 1.97)	0.79	0.88 (0.60 to 1.28)	0.50	0.93 (0.67 to 1.29)	0.67
Orientation (0–6)	5.8 (0.4)	5.8 (0.5)	5.9 (0.4)	1.67 (0.67 to 4.13)	0.27	1.16 (0.60 to 2.24)	0.66	1.30 (0.75 to 2.24)	0.35
Abstraction (0–2)	1.7 (0.6)	1.5 (0.7)	1.6 (0.6)	1.03 (0.51 to 2.10)	0.94	1.67 (1.08 to 2.58)	0.022	1.45 (0.99 to 2.13)	0.053
*MoCA*†									
Normal (%)	30 (78.9%)	68 (73.9%)	1080 (74.2%)	0.93 (0.40 to 2.16)	0.86	1.08 (0.65 to 1.78)	0.77	1.04 (0.67 to 1.61)	0.87
Mild cognitive impairment or dementia (%)	8 (21.1%)	24 (26.1%)	376 (25.8%)						

Variable	GD-causing variants (group 1) n=44	E326K and T369M carriers (group 2) n=98	Non-carriers (group 3) n=1584	Beta* (95% CI) Group 1 vs 3	P value* Group 1 vs 3	Beta* (95% CI) Group 2 vs 3	P value* Group 2 vs 3	Beta* (95% CI) Groups 1 and 2 vs 3	P value* Groups 1 and 2 vs 3
MoCA total score	25.9 (2.7)	25.0 (3.3)	25.2 (3.5)	0.28 (−0.78 to 1.33)	0.61	−0.37 (−1.07 to 0.32)	0.29	−0.18 (−0.77 to 0.41)	0.54

Scores in first three columns are means (SD) except for categorised MoCA, which is N (%). ORs are calculated such that increasing values (and increasing odds) are associated with worse cognition.

*Adjusted for age, gender, disease duration and years of education.

†Mild cognitive impairment classification based on education adjusted score of 22–23 and dementia based on a MoCA education adjusted test score of < 22.

GD, Gaucher’s disease.

**Table 4:** Cognitive and behavioural impairment in the *GBA*-associated Parkinson’s disease cohort based on the Movement Disorder Society Unified Parkinson’s disease rating scale part 1 scores dichotomised at 1 or above

Variable	GD-causing variants (group 1) n=44	E326K and T369M carriers (group 2) n=98	Non-carriers (group 3) n=1584	OR^*^ (95% CI) Group 1 vs 3	P value^*^ Group 1 vs 3	OR^*^ (95% CI) Group 2 vs 3	P value^*^ Group 2 vs 3	OR^*^ (95% CI) Groups 1 and 2 vs 3	P value^*^ Groups 1 and 2 vs 3
Cognitive impairment	16 (36.4%)	36 (36.7%)	620 (39.6%)	0.96 (0.51 to 1.81)	0.89	0.93 (0.61 to 1.43)	0.74	0.94 (0.65 to 1.35)	0.73
Hallucinations and psychosis	6 (13.6%)	8 (8.2%)	137 (8.8%)	1.70 (0.70 to 4.13)	0.24	1.00 (0.47 to 2.11)	1	1.21 (0.68 to 2.17)	0.52
Depressed mood	17 (38.6%)	35 (35.7%)	574 (36.6%)	1.01 (0.54 to 1.87)	0.98	0.95 (0.62 to 1.46)	0.83	0.97 (0.68 to 1.39)	0.87
Anxious mood	22 (50.0%)	46 (46.9%)	769 (49.1%)	0.95 (0.52 to 1.75)	0.88	0.90 (0.60 to 1.37)	0.63	0.92 (0.65 to 1.30)	0.63
Apathy	13 (29.5%)	38 (38.8%)	464 (29.7%)	0.94 (0.48 to 1.82)	0.85	1.48 (0.97 to 2.26)	0.069	1.29 (0.90 to 1.86)	0.16
Impulse control disorders	2 (4.5%)	9 (9.4%)	82 (5.2%)	0.66 (0.15 to 2.83)	0.58	1.96 (0.95 to 4.06)	0.070	1.46 (0.75 to 2.83)	0.26

Data are presented in the table as n (%).

* Adjusted for age, gender and disease duration.

GD, Gaucher’s disease.

### Demographic and clinical features

In 142 PD cases who had a variant detected in the *GBA* gene (all cases), mean age was 65.6 years (SD 10.1) years, mean disease duration was 1.3 (SD 0.9) years and 93 (65.5 %) of these were male. Other demographic features of these cases classified by genotype (excluding cases with variants of unknown significance and those with unusual presentation) compared with non-carriers are detailed in table 2.

Cases who carried GD-causing variants in the *GBA* gene were younger (mean age 62.9, SD 12.3 years) compared with non-carriers (mean age 67.6 years, SD 9.2 years) (P<0.001), had an earlier age at onset (mean 59.7 years, SD 12.8 years) compared with non-carriers (mean 64.4 years, SD 9.7 years) (P=0.002) and were at a more advanced Hoehn and Yahr stage compared with non-carriers (P=0.02) when adjusted for age and disease duration (table 2). This was also reflected in greater medication requirements at baseline in those with GD-causing variants (levodopa-equivalent daily dose (LEDD) 370 mg/day, SD 219 mg/day) compared with non-carriers (LEDD 292 mg/day, SD 206 mg/day) (P=0.03). The postural instability gait difficulty (PIGD) motor subtype was the most common phenotype in those with GD-causing variants compared with those with non-carriers (adjusted P=0.04) reflecting more axial involvement and less tremor. E326K and T369M carriers when analysed together with carriers of GD-causing variants also showed PIGD as the most common motor subtype compared with non-carriers (adjusted P=0.03). The prevalence of anxiety (adjusted P=0.44), depression (adjusted P=0.28) and cognitive impairment (adjusted P=0.84) was not different between those with GD-causing variants (and those carrying the E326K, T369M variants) and non-carriers.

There was no statistically significant difference in the prevalence of RBD symptoms between those with mutations in the *GBA* gene and non-carriers (table 2). However, olfactory loss was more prevalent in those carrying GD causing mutations than non-carriers (P=0.047).

### Cognitive profiles based on MoCA testing

There was no difference in cognition as measured by average MoCA test score or proportion of patients with dementia between those with and without pathogenic *GBA* mutations (table 3). There was no difference in cognition between possibly atypical PD cases carrying *GBA* mutations and PD non-carriers. (online supplementary table 1). Compared with non-carriers, those with GD-causing variants had lower scores on the naming domain of the MoCA test when adjusted for age, gender, disease duration and years of education (adjusted P=0.01), which did not survive correction for multiple testing. There were no significant group differences between those with GD-causing variants, E326K and T369M carriers and non-carriers in the scores of other domains of cognitive function such as attention (adjusted P=0.18), orientation (adjusted P=0.28), language (adjusted P=0.37) and delayed recall (adjusted P=0.67). However, E326K and T369M carriers had significant lower scores on the abstraction domain compared with those carrying GD-causing variants (adjusted P=0.02).

10.1136/jnnp-2017-317348.supp1 Supplementary file 1

Repeating the analyses for motor features, cognitive impairment and behavioural domains did not change the results.

Cases with p.L444P mutations did not show any difference in total MoCA test scores or any of the subdomains except naming (with a very small absolute difference) with non-carriers (online supplementary table 1). Cases with p.L444P mutations did not show any difference in any of the subdomains or total MoCA test scores compared with p.N370S mutation carriers (online supplementary table 2).

The data available from the answers to the six questions from part 1 of the MDS-UPDRS scale provide a measure of the impact of cognitive dysfunction and complex behaviours on the ability to perform activities of daily living as shown in table 4.

Finally, the imputed analyses showed no qualitative differences between the imputed and complete case results (online supplementary tables 1-7).

## Discussion

To our knowledge, this is the largest single cohort study examining the influence of *GBA* mutations in newly diagnosed PD in terms of age at onset, motor phenotype and cognitive functions. We found that *GBA* mutations affected age at onset of PD and the motor phenotype, with no apparent effect on any aspect of cognition at the early stages of the disease process (average disease duration 1.5 years). We excluded patients with dementia at baseline as this is necessary to define PD as per Queen Square Brain Bank criteria.^21^ This means that patients with *GBA*-associated DLB are not included in this study.

2.5% of our cohort carried one or more pathogenic mutations in the *GBA* gene. This is lower than the proportion of cases found in a meta-analysis of European studies (5.5%)^26^ and a North American study (4.4%)^18^ but closer to the rate of *GBA* mutations found in a meta-analysis of data from studies in the Chinese population (3.6%).^27^ Higher proportions in other studies probably reflect data from enriched populations due to a referral bias to specialist centres for genetic testing. Our study reflects data collected from the National Health Service hospitals that normally provide routine healthcare in the UK, reflecting a diverse unbiased population pool in the community. L444P was the most common pathogenic *GBA* mutation (1.6%) in patients with PD in our study. This is in keeping with the results of a previous study from China that showed L444P as the most common mutation detected in *GBA* in cases with PD (n=402).^27^ This is not surprising given that L444P is a common pan-ethnic mutation while N370S is the most common mutation in Jewish populations.^4^

Some have suggested that *GBA* mutation carriers are more likely to have a family history of PD^28^ but we did not find any significant differences in the proportion of people with or without *GBA* mutations and a family history of PD among first-degree or second-degree relatives. We did not directly ask our participants whether they had Jewish ancestry but <1% reported that their religion was Judaism even though we included study centres in UK populations with large Jewish communities: North London, Manchester and Glasgow.

We found E326K in 4.5% of our patients with PD, which is close to the 4.7% figure reported in a cohort of similar size (n=1369) from the USA.^18^ The background control frequency of E326K in the UK has been reported to be approximately 2.5%. In our series, E326K was not associated with early-onset PD. One case in our series was homozygous for the E326K variant and had an age of onset 64.6 years and had similar motor features to E326K and T369M heterozygote carriers (group 2). This homozygous E326K case did not have GD, confirming that homozygosity for this allele does not cause GD, as described by others.^7^ One recent study reported the frequency of the E326K polymorphism in their cohort of patients with PD was no different from their controls, suggesting that E326K was not pathogenic for PD.^29^ However, results from a meta-analysis of data from five recent PD genome-wide association studies indicated that E326K is a susceptibility allele for PD.^30^

We found that our cases with *GBA* mutations were younger at diagnosis compared with non-carriers by about 5 years. Our data are consistent with a study from North America that reported similar findings.^31^ Another study from Germany similarly showed the age of onset in *GBA* mutation carriers was on average 6 years earlier compared with non-carriers,^12^ and a previous meta-analysis of several studies showed *GBA* mutation carriers had an earlier age of onset of around 4.6 years compared with PD patients without *GBA* mutations.^32^

We confirm previous reports suggesting that *GBA* variants can influence the motor phenotype of patients with PD.^32^ Those with *GBA* mutations in our study were at a more advanced Hoehn and Yahr stage, even when adjusted for disease duration, compared with non-carriers, which is consistent with previous reports.^11^ However, the UPDRS motor scores in our study were not significantly different between the groups, although all our patients were assessed ‘on’ medication. Those with *GBA* mutations (including E326K and T369M) were more likely to have the PIGD phenotype compared with non-carriers and had higher dopaminergic therapy requirements compared with non-carriers, possibly explaining the lack of difference in UPDRS motor scores. This was not reported by Winder-Rhodes *et al* in their mutation carriers (n=9),^11^ possibly because differences can be difficult to detect with small numbers of patients.

Earlier reports found that *GBA* variants are associated with greater impairment in working memory, executive function and visuospatial abilities^18^ and *GBA* mutations confer a greater risk of dementia during the course of PD.^17^

The development of therapeutic agents that exploit the key pathological pathways in *GBA*-associated PD include the small molecular chaperone Ambroxol,^33^ which crosses the blood–brain barrier when administered orally, have been shown to increase *GBA* enzyme activity in primate models.^34^ Therefore, if such therapeutic strategies were to become available for humans, we would need to identify a window of opportunity before the onset of dementia in *GBA*-associated PD where such interventions would have the maximum impact. Those earlier studies were reporting differences in cognitive function at an average disease duration of 12–15 years,^16 17^ whereas we are presenting baseline data.

Our findings are consistent with other previous studies that describe patients at this relatively early stage of the disease course (<3.5 years from disease onset) in which they did not find differences in cognitive function between patients carrying *GBA* mutations and non-carriers.^9 11 35^ Importantly, in those studies where cognitive scores were assessed longitudinally, patients carrying *GBA* mutations only begin to show divergence in cognitive performance around 36 months after motor onset (or diagnosis).^9 11^

The cognitive measures used in our study are restricted to the MoCA test. It is a widely validated test with sensitivity to detect changes in several cognitive domains for screening all levels of cognition in PD.^23^ Sensitivity and specificity for PD dementia using MoCA are 81%–82% and 75%–95%, respectively. Sensitivity and specificity for MCI in PD are 83%–90% and 53%–75%, respectively.^23^ We did not detect any differences at early-stage disease between those with pathogenic mutations in *GBA* and non-carriers in the attention, orientation, language, delayed recall and visuospatial domains using the MoCA test. While the MoCA has good sensitivity for detecting dementia and is a test of global cognition, subtle differences in individual cognitive domains may not be detected using the MoCA test even where they exist. Future studies could specifically address this issue with more sensitive neuropsychological measures in patients with specific mutations in order to detect more subtle cognitive deficits that may be found at an earlier stage.

Previous reports from genotype–phenotype correlative studies have suggested that those with p.L444P mutations (which are thought to cause a more severe GD phenotype) have a greater degree of cognitive impairment compared with patients with p.N370S mutations (which are thought to cause a less severe GD phenotype).^8^ We did not detect any differences in the cognitive performance on the MoCA test at this early stage in the disease in patients with these 2 types of mutations.

Besides cognitive impairment, previous reports suggest more severe anxiety and depression in *GBA-*associated PD compared with those with sporadic PD.^35 36^ The prevalence of anxiety in our study was not significantly different in pathogenic GD mutation carriers compared with non-carriers, and the same was true for depression Furthermore, we did not find any significant differences between the prevalence of neurobehavioural and psychiatric symptoms such as psychosis between the three groups of patients in our study. This may relate to the relatively early stage of disease in our cohort with a relatively shorter disease duration as psychosis in PD typically occurs in the later stages of the disease and after several years of treatment^37 38^ and differences may emerge with longer follow-up duration.

Strengths of our study include comprehensive screening of the *GBA* gene as opposed to selective mutation detection; the size of the study and the representative population base. Weaknesses of our paper include the cross-sectional family history data and the use of a brief cognitive screening tool.

## Conclusion

We show that patients with PD and *GBA* mutations are different from *GBA* mutation non-carriers. *GBA* mutation carriers had an earlier age of onset, more commonly had the PIGD phenotype and had more severe motor impairment. In line with previous studies, global cognition scores did not differ between *GBA* mutation carriers and non-carriers at early stages in the disease process. Follow-up data emerging from this study will be used to reassess cognitive function in *GBA* mutation carriers at a later stageas well as the development of any neuropsychiatric and behavioural problems.
