# Supplementary material for: Features of GBA-associated Parkinson’s disease at presentation in the UK Tracking Parkinson’s study
Source: J Neurol Neurosurg Psychiatry. 2018 Jan 29;89(7):702–9. doi: 10.1136/jnnp-2017-317348 (PMC6031283; doi:10.1136/jnnp-2017-317348)
Supplement: Supplementary file 1 [file jnnp-2017-317348supp001.pdf]

Supplementary Table 1. Cognitive impairment in the *GBA*-associated PD cohort classified by subdomain based on the MoCA test. Odds ratios are calculated such that increasing values (and increasing odds) are associated with worse cognition. Linearity transformations of continuous confounders taken from whole group analysis.

**Variable**	**p.L444P carriers only (group 1a) n=27**	**Non-carriers (group 3) n=1584**	**Atypical cases with any *GBA* mutations (group 4) n=23**	**OR[a] (95% CI) Group 1a vs. 3**	**p-value[a] Group 1a vs. 3**	**OR[a] (95% CI) Group 4 vs. 3**	**p-value[a] Group 4 vs. 3**
Visuospatial	4.2 (1.2)	4.3 (1.0)	4.3 (0.9)	1.65 (0.74, 3.68)	0.22	1.27 (0.56, 2.90)	0.56
Attention	5.2 (1.0)	5.3 (1.0)	5.4 (0.8)	1.56 (0.73, 3.31)	0.25	1.08 (0.49, 2.42)	0.84
Language	2.6 (0.5)	2.4 (0.8)	2.7 (0.6)	1.10 (0.51, 2.34)	0.81	0.54 (0.21, 1.42)	0.21
Naming	2.8 (0.4)	2.9 (0.3)	2.9 (0.3)	4.65 (1.58, 13.65)	0.005	2.41 (0.67, 8.60)	0.18
Recall	2.8 (1.8)	2.6 (1.6)	3.2 (1.6)	1.75 (0.80, 3.81)	0.16	0.77 (0.37, 1.58)	0.47

Orientation	5.9 (0.3)	5.9 (0.4)	5.8 (0.5)	1.58 (0.45, 5.51)	0.47	1.49 (0.42, 5.22)	0.53
Abstraction	1.7 (0.5)	1.6 (0.6)	1.7 (0.6)	1.11 (0.46, 2.71)	0.82	1.18 (0.46, 3.05)	0.73
MoCA †							
Normal (%)	19 (79.2%)	1080 (74.2%)	17 (81.0%)	1.36 (0.48, 3.87)	0.56	0.74 (0.24, 2.33)	0.61
MCI or dementia (%)	5 (20.8%)	376 (25.8%)	4 (19.1%)				
**Variable**	**p.L444P carriers only (group 1a) n=27**	**Non-carriers (group 3) n=1584**	**Atypical cases with *GBA* mutations (group 4) n=23**	**Beta[a] (95% CI) Group 1a vs. 3**	**p-value[a] Group 1a vs. 3**	**Beta[a] (95% CI) Group 4 vs. 3**	**p-value[a] Group 4 vs. 3**
MoCA total score	25.8 (2.9)	25.2 (3.5)	26.3 (3.4)	-0.27 (-1.61, 1.06)	0.69	0.86 (-0.56, 2.28)	0.24

Data are presented in the table as mean (sd). OR= odds ratio, CI= confidence interval, MoCA= Montreal cognitive assessment

[a]Adjusted for age, gender, disease duration and years of education

Supplementary Table 2. Cognitive impairment in the *GBA*-associated PD cohort classified by subdomain based on the MoCA test. Odds ratios are calculated such that increasing values (and increasing odds) are associated with worse cognition. Individuals with both mutations (n=2) were put into the p.L444P group. Linearity transformations of continuous confounders taken from whole group analysis.

**Variable**	**p.L444P carriers only (group 1a) n=27**	**p.N370S carriers only (group 1b) n=8**	**OR[a] (95% CI) Group 1a vs. 1b**	**p-value[a] Group 1a vs. 1b**
Visuospatial	4.2 (1.2)	4.4 (0.9)	1.99 (0.20, 19.99)	0.56
Attention	5.2 (1.0)	5.6 (0.7)	4.11 (0.46, 36.45)	0.20
Language	2.6 (0.5)	2.6 (0.7)	3.32 (0.38, 29.06)	0.28
Naming	2.8 (0.4)	3.0 (0)	NA*	NA*
Recall	2.8 (1.8)	2.4 (1.3)	5.01 (0.84, 30.04)	0.078
Orientation	5.9 (0.3)	5.5 (0.8)	0.97 (0.06, 15.13)	0.98
Abstraction	1.7 (0.5)	1.4 (0.7)	0.73 (0.06, 8.70)	0.80
MoCA †				
Normal (%)	19 (79.2%)	5 (62.5%)	0.77 (0.09, 6.78)	0.82
MCI or dementia (%)	5 (20.8%)	3 (37.5%)		
**Variable**	**p.L444P carriers only (group 1a) n=27**	**p.N370S carriers only (group 1b) n=8**	**Beta[a] (95% CI) Group 1a vs. 1b**	**p-value[a] Group 1a vs. 1b**
MoCA total score	25.8 (2.9)	25.3 (2.7)	-0.80 (-3.63, 2.02)	0.56

Data are presented in the table as mean (sd). OR= odds ratio, CI= confidence interval, MoCA= Montreal cognitive assessment

[a]Adjusted for age, gender, disease duration and years of education

*Perfect prediction cannot estimate effect no variation in p.N370S group

Supplementary Table 3. Demographic and clinical features of the PD cohort classified by *GBA* mutation carrier status **(this is imputed analysis of data presented in Table 2)**.

Variable	Adjusted[a] p-value Group 1 vs. Non-carriers (Group 3)	Adjusted[a] p-value Group 2 vs. Non-carriers (Group 3)	Adjusted[a] p-value Group 1 and Group 2 vs. Non-carriers (Group 3)
HY stage 0-1.5 (%)	0.015	0.81	0.14
2 or 2.5 (%)			
3 + (%)			
UPDRS 3	0.23[b]	0.93[b]	0.57[b]
LEDD (mg/day)	0.029	0.17	0.022
Education - ≤ 12 yrs	0.51	0.86	0.83
Motor subtype TD			
PIGD	0.12	0.25	0.072
Mixed	0.073	0.56	0.15
Anxiety	0.40	0.90	0.57
Depression	0.95	0.18	0.30
QOL score	0.98	0.34	0.43
Family h/o PD 1st degree	0.80	0.19	0.23
2nd degree	0.55	0.062	0.22
Any	0.86	0.047	0.084

Data are shown as mean and standard deviation unless expressed otherwise. GD= Gaucher's disease, HY= Hoehn Yahr stage, UPDRS 3= Unified Parkinson's disease rating scale Part 3, LEDD= levodopa equivalent daily dose, QOL = quality of life based on PD-Q8 scale

[a]Adjusted for age, gender and disease duration (except where otherwise noted)

[b]Adjusted for age, gender, disease duration and LEDD

Supplementary Table 4 (this is imputed analysis of data presented in Table 3). Cognitive impairment in the *GBA*-associated PD cohort classified by subdomain based on the MoCA test, data is mean (sd) except where stated

Variable	Adjusted[a] Odds ratio (95% CI) Group 1 vs. Non-carriers (Group 3)	Adjusted[a] p-value Group 1 vs. Non-carriers (Group 3)	Adjusted[a] Odds ratio (95% CI) Group 2 vs. Non-carriers (Group 3)	Adjusted[a] p-value Group 2 vs. Non-carriers (Group 3)	Adjusted[a] Odds ratio (95% CI) Group 1 and Group 2 vs. Non-carriers (Group 3)	Adjusted[a] p-value Group 1 and Group 2 vs. Non-carriers (Group 3)
Visuospatial	1.28 (0.70, 2.33)	0.42	1.11 (0.74, 1.67)	0.62	1.16 (0.82, 1.64)	0.41
Attention	1.19 (0.66, 2.11)	0.56	1.28 (0.87, 1.87)	0.21	1.25 (0.90, 1.73)	0.18
Language	0.71 (0.38, 1.34)	0.29	1.33 (0.89, 1.97)	0.17	1.10 (0.78, 1.54)	0.59
Naming	2.74 (1.15, 6.55)	0.023	1.04 (0.49, 2.22)	0.92	1.46 (0.82, 2.61)	0.20
Recall	1.02 (0.57, 1.83)	0.95	0.90 (0.63, 1.30)	0.58	0.94 (0.68, 1.28)	0.68
Orientation	1.47 (0.60, 3.62)	0.40	1.02 (0.53, 1.97)	0.95	1.15 (0.67, 1.97)	0.62
Abstraction	0.91 (0.44, 1.86)	0.79	1.55 (1.01, 2.38)	0.046	1.33 (0.91, 1.93)	0.14
MoCA †						
Normal vs. MCI or dementia	1.10 (0.49, 2.46)	0.81	1.10 (0.67, 1.80)	0.71	1.10 (0.72,1.68)	0.66
**Variable**	**Beta[a] (95%**	**p-value[a]**	**Beta[a] (95%**	**p-value[a]**	**Beta[a]**	**p-value[a]**

	CI) Group 1 vs. 3	Group 1 vs. 3	CI) Group 2 vs. 3	Group 2 vs. 3	(95% CI) Groups 1 & 2 vs. 3	Groups 1 & 2 vs. 3
MoCA total score	-0.10 (-1.09, 0.89)	0.84	-0.32 (-0.99, 0.34)	0.34	-0.25 (-0.82, 0.31)	0.38

MoCA= Montreal cognitive assessment test

† Mild cognitive impairment classification based on Montreal cognitive assessment (MoCA) education adjusted test score of 22-23, and dementia based on a MoCA education adjusted test score of <22, [a]Adjusted for age, gender, disease duration and years of education

Supplementary Table 5 (this is imputed analysis of data presented in Table 4).

Cognitive and behavioural impairment in the *GBA*-associated PD cohort based on the MDS-UPDRS part 1 scores dichotomised at 1 or above, data is n (%)

Variable	Adjusted[a] Odds ratio (95% CI) Group 1 vs. Non carriers (Group 3)	Adjusted[a] p-value Group 1 vs. Non carriers (Group 3)	Adjusted[a] Odds ratio (95% CI) Group 2 vs. Non carriers (Group 3)	Adjusted[a] p-value Group 2 vs. Non carriers (Group 3)	Adjusted[a] Odds ratio (95% CI) Group 1 and Group 2 vs. Non carriers (Group 3)	Adjusted[a] p-value Group 1 and Group 2 vs. Non carriers (Group 3)
Cognitive impairment	0.96 (0.51, 1.81)	0.90	0.93 (0.61, 1.43)	0.75	0.94 (0.66, 1.35)	0.74
Hallucinations and psychosis	1.69 (0.69, 4.11)	0.25	0.99 (0.47, 2.09)	0.98	1.20 (0.67, 2.15)	0.53
Depressed mood	1.01 (0.54, 1.87)	0.98	0.95 (0.62, 1.46)	0.83	0.97 (0.68, 1.39)	0.87
Anxious mood	0.96 (0.52, 1.76)	0.89	0.91 (0.60, 1.37)	0.65	0.92 (0.65, 1.31)	0.65
Apathy	0.93 (0.48, 1.81)	0.84	1.47 (0.97, 2.25)	0.072	1.29 (0.90, 1.85)	0.17
Dopamine dysregulation syndrome a	0.67 (0.16, 2.85)	0.58	1.96 (0.95, 4.06)	0.070	1.47 (0.76, 2.85)	0.26

MDS-UPDRS= Movement Disorder Society- Unified Parkinson’s disease rating scale

[a]Adjusted for age, gender and disease duration

Supplementary Table 6 (this is imputed analysis of data presented in Supplementary Table 1). Cognitive impairment in the *GBA*-associated PD cohort classified by subdomain based on the MoCA test. Odds ratios are calculated such that increasing values (and increasing odds) are associated with worse cognition. Linearity transformations of continuous confounders taken from whole group analysis.

**Variable**	**OR[a] (95% CI) Group 1a (p.L444P) vs. 3 (non-carriers)**	**p-value[a] Group 1a (p.L444P) vs. 3 (non-carriers)**
Visuospatial	1.53 (0.71, 3.28)	0.28
Attention	1.47 (0.71, 3.04)	0.29
Language	0.93 (0.44, 1.97)	0.85
Naming	3.85 (1.33, 11.13)	0.013
Recall	1.46 (0.69, 3.12)	0.32
Orientation	1.41 (0.41, 4.88)	0.59
Abstraction	0.99 (0.41, 2.39)	0.98
MoCA †		
Normal vs. MCI or dementia	1.71 (0.65, 4.48)	0.28
**Variable**	**Beta[a] (95% CI) Group 1a (p.L444P) vs. 3 (non-carriers)**	**p-value[a] Group 1a (p.L444P) vs. 3 (non-carriers)**
MoCA total score	-0.78 (-2.04, 0.48)	0.22

Data are presented in the table as mean (sd). OR= odds ratio, CI= confidence interval,

MoCA= Montreal cognitive assessment

[a]Adjusted for age, gender, disease duration and years of education

Supplementary Table 7 (this is imputed analysis of data presented in Supplementary Table 2). Cognitive impairment in the *GBA*-associated PD cohort classified by subdomain based on the MoCA test. Odds ratios are calculated such that increasing values (and increasing odds) are associated with worse cognition. Individuals with both mutations (n=2) were put into the p.L444P group. Linearity transformations of continuous confounders taken from whole group analysis.

**Variable**	**OR[a] (95% CI) Group 1a (p.L444P) vs. 1b (p.N370S)**	**p-value[a] Group 1a (p.L444P) vs. 1b (p.N370S)**
Visuospatial	2.09 (0.24, 18.33)	0.50
Attention	3.90 (0.49, 30.82)	0.20
Language	2.67 (0.33, 21.73)	0.36
Naming	NA*	NA*
Recall	2.60 (0.50, 13.69)	0.26
Orientation	0.70 (0.05, 9.53)	0.79
Abstraction	0.68 (0.06, 7.76)	0.76
MoCA †		
Normal vs. MCI or dementia	0.90 (0.10, 8.05)	0.92
**Variable**	**Beta[a] (95% CI) Group 1a (p.L444P) vs. 1b (p.N370S)**	**p-value[a] Group 1a (p.L444P) vs. 1b (p.N370S)**
MoCA total score	-1.37 (-4.61, 1.87)	0.39

Data are presented in the table as mean (sd). OR= odds ratio, CI= confidence interval, MoCA= Montreal cognitive assessment

[a]Adjusted for age, gender, disease duration and years of education

*Perfect prediction cannot estimate effect no variation in p.N370S group
